# Comparison of Some Behavioural Responses in Budgerigars (*Melopsittacus undulatus*) Raised in Cages Enriched with Coloured LED Lights

**DOI:** 10.3390/ani12182454

**Published:** 2022-09-16

**Authors:** Demirel Ergun, Atilla Taskin

**Affiliations:** 1 Graduate School of Science, Kirsehir Ahi Evran University, Kirsehir 40100, Türkiye; 2 Department of Animal Science, Faculty of Agriculture, Kirsehir Ahi Evran University, Kirsehir 40100, Türkiye

**Keywords:** budgerigar, neophobia, welfare assessment

## Abstract

**Simple Summary:**

In this study, the environment of hand-raised budgerigars in captivity (people’s homes) was simulated. We investigated the effect of coloured LED lights in addition to natural light on the behaviour of the birds. For birds raised by humans, the light in houses is significantly different from the lighting conditions in a bird’s natural habitat. This may lead to behavioural and health problems in birds, adversely affecting their wellbeing. This study investigated how white, blue, yellow, and red LED lights applied at the beginning of the dark cycle of the photoperiod affected the behaviours of budgerigars. The results indicated that when offered the opportunity, the birds chose yellow light. The open field tests (OFT I and II) were used to assess the locomotion and exploratory behaviour of birds. The results of the OFT I test, which was performed with an unfamiliar object, indicated that the group experiencing yellow light was more self-confident. The birds in cages with blue light felt more comfortable and displayed more social behaviour. People will be able to use this new information to improve the living conditions of their pet budgerigars.

**Abstract:**

This study examined the effects of exposure to coloured LED lights on some behavioural responses, food and water consumption, and live weight in budgerigars kept in captivity using behavioural tests to compare different lighting conditions. Budgerigars’ feeding, comfort, social, fear, and resting behaviours and behavioural reactions to a new environment were studied. Twelve male birds were assigned to four groups, with three birds in each group. They were provided with food and water ad libitum in wire cages at 25 ± 2 °C in a room. The study was conducted with 10 h of natural light, 6 h of LED light (white, blue, yellow, and red LED lights), and 8 h of darkness. Their behaviours in the cages were observed. Home cage avoidance (HCA) and two open field tests (OFT) were performed. No difference was observed in the LW and food consumption, but the blue and yellow groups consumed less water. The blue group displayed more comfort and social behaviours. In the HCA test, the red group demonstrated higher reactions. Furthermore, the birds in the yellow group stayed closer to the centre of the platform in the OFT I test, and they chose the compartment simulating their familiar environment in the OFT II test. Consequently, it was concluded that first yellow and then blue lights may be used to prevent stress-related problems for these birds. Our findings could be used to improve the hand-rearing conditions of captive budgerigars.

## 1. Introduction

The budgerigar (*Melopsittacus undulatus*) is a small parrot species (Psittaciformes) that originated on the continent of Australia [1]. Due to its striped feathers, it was initially reported as a transitional species between the *Neophema* and the *Pezoporus* [2]. However, phylogenetic studies based on DNA sequences revealed that the species, *M. undulatus*, is closely related to the red parrot and the fig parrot [3]. The first budgerigar domestication efforts were reported in England in the 1850s and expanded to the European continent [4,5]. 

Today, the rate of owning pets has increased. Having pets (dogs, cats, or birds) may have positive effects on mental and physical health (such as interacting with people, being outside, and exercise) [6,7,8]. *M. undulatus* is renowned as an intelligent and social bird. They are popular pets, as it is simple to care for them, they mimic words, have the ability to vocalize, and have vibrant and appealing feather hues [9,10]. 

Birds have tetrachromatic colour vision mediated by single cones sensitive to ultraviolet, short, medium, and long wavelengths [11]. Birds use spectral information for circadian control, magnetic orientation, phototaxis and to distinguish the colours of important objects such as food. Additionally, colour discrimination and constancy are fine in diurnal birds [12]. The budgerigar has four cone pigments with λ_max_ at 370, 445, 508, and 565 nm as shown by microspectrophotometry [13]. The spatial and colour vision of budgerigars declines with decreasing light intensity and is finally lost [14]. Studies of animal colour vision often assume that cones adapt independently to background light (for example, green vegetation), which is called von Kries adaptation [15]. 

With the variety of light sources today, the utilization of light for multiple purposes has also increased. Thus, light pollution has arisen, and light has become a stressor, either directly or indirectly [16]. Some studies on the biological effect of artificial light on organisms showed that artificial light at night has negative effects on many creatures [16,17]. If light restrictions are not achievable, then it is necessary to determine the wavelength (colour) at which living beings would be least harmed. The use of LED lights with a light spectrum, colour composition, and adjustable light technology might reduce the possible stress impact of light on flora and fauna [18].

There have been many studies on the effect of light wavelengths on many organisms including birds [19,20,21]. In general, for birds, it has been reported that light with long wavelengths penetrates the brain more easily and has more of an observable effect than short wavelengths [22]. It was reported that, in addition to environmental factors, the genetic structure was also effective in shaping behaviours in birds [23]. For example, a study on black-headed buntings (*Emberiza melanocephala*) and baya weavers (*Ploceus philippinus*) reported that the birds perceived blue light as night and red light as day [24]. Furthermore, compared to the control group, the stress hormone (corticosterone) levels of birds that nested in places with white light and near red lights was reportedly higher [25]. Coloured lights were also reported to have affected the orientation of birds [26]. Red lights installed on high wind turbines were reported to have compromised the navigation of *Zosterops lateralis* throughout their migratory route, resulting in a high mortality rate [27]. Similarly, yellow light has been reported to have negatively affected the orientation behaviour of *Erithacus rubecula*, while artificial light in the near-blue spectrum had a strong negative effect on bird groups in their natural habitat, including seabirds [28,29].

Birds that are bred as pets may be exposed to the possible negative effects of light more than wild birds [30]. The species of *M. undulatus* is one of the best examples of this condition. Budgerigars that have been removed from their native habitat to be domesticated are known to experience various challenges in adapting to their new environments. One of the major challenges they have in their new environments is ambient light, which influences their circadian rhythm. The primary issue is that the bird spends the time that it would normally spend in the dark in its natural environment in the light, depending on human activities. Anomalies including stress, obesity, and behavioural disorders develop in birds due to this condition [30,31].

Many tests are used for behavioural observations in captive birds. Some of these include observation in cage, open field (can be applied with familiar and unfamiliar object), and home cage avoidance (HCA) tests. The HCA test is one of the methods used to predict fear in birds. The primary idea is to gather information regarding fear behaviour by recording the responses of birds to unfamiliar objects. This test may provide insight into fear behaviour without allowing the birds to escape the cage [32]. Interested behaviours are the fundamental components that build the feeling of discovery in birds. These are among the behaviours required for avian survival. The OFT test is used to assess an individual’s degree of reaction and exploration when placed in a new environment [33]. Numerous bird species have been known to exhibit neophobic behaviours, such as a reluctance to approach unfamiliar objects. This affects avian behaviours to different degrees [34]. In open field tests, birds had a more acute reaction to novelty. This acute reaction might cause a delay in overcoming initial fear and taking the first step toward novelty. Furthermore, depending on the extent of the novelty-induced fear, birds may reduce their activity even further or display responsive behaviours such as fluttering and jumping [35]. In general, birds take longer to explore and approach unfamiliar objects than they do to reapproach previously seen objects [36]. 

The significance of light for budgerigar health, breeding, behaviour, and animal welfare has been shown in numerous studies [37,38]. Understanding the behaviours of animals contributes not only to assessing their present condition but also to predicting their future condition [39]. Birds’ behaviours that are different from what is normal or expected may indicate stress. The duration of light exposure and the wavelength of light affect the severity of stress [40]. It has been reported that the duration, amount, and wavelength of light in birds affected mating [41], vocalization [42], sleep [43], species-specific behaviours, and anxiety levels [44]. The visible light spectrum is roughly ranked, with blue and red at opposite ends and yellow in the middle. In white light, the situation is much different. White light is not actually a colour, so it is not possible to talk about a single wavelength in white light. To obtain white light, a mixture of wavelengths, or different colours, is required. When all wavelengths of the visible light spectrum reach the eye simultaneously, it is perceived as white. For this reason, blue-, red-, yellow- and white-coloured LEDs were used in this study. In the study, the effects of coloured lights on the changes observed in the behavioural responses of birds including fear, adapting to a new environment, feeding, comfort, sociability, and resting were recorded. The study describes the relationship between the welfare and behaviours of budgerigars and coloured lights.

## 2. Materials and Methods

### 2.1. Animals, and Management

Twelve sexually mature male birds (*M. undulatus*; age 12 months; body mass 44.78 ± 8.27 g (*p* = 0.182), blue cere, green plumage) were used for the experiments (*F*-test). The birds were procured from a domestic pet shop licensed by the Ministry of Agriculture and Forestry. This study was carried out from March to April 2022. Male birds were used in the study, since the stress effect of the sexual cycle is greater in female birds, male birds have better vocalization skills than female birds, and male birds are preferred by breeders [45,46]. 

The study was conducted in a controlled setting with a temperature of 25 ± 2 °C, a relative humidity of 55 ± 5%, and a noise level of 35–40 dB. A total of four commercial cages made of easy-to-clean metal, measuring 60 × 40 × 30 cm, one for each group, were utilized in the study [30,47,48]. The study was carried out with three birds in each of the four groups, organized homogeneously after the quarantine period had expired (at the end of the 15 days).

In the study, both commercially produced budgerigar feed (Table 1) and water (Table 2) intended for human consumption were utilized. The birds were provided with food and water ad libitum. In addition, the daily water- and food-consumption amounts per bird were determined for the experimental groups.

### 2.2. Enrichment with LED light

**Figure 1:**
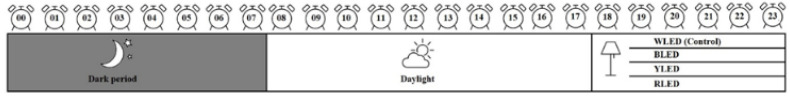
The daily photoperiod and additional LED light program. WLED (control): the group was exposed to white LED light (400–740 nm), representing today’s human home environment. BLED: the group was exposed to blue LED light (450–490 nm). YLED: the group was exposed to yellow LED light (560–590 nm). RLED: the group was exposed to red LED light (635–700 nm).

The cages were placed in four compartments (for blue, white, yellow, and red lights) made of expanded polystyrene (TS7316- EN13163), the front parts of which were open and opaque, specially designed for the study with dimensions of 120 × 60 × 60 cm (Figure 1). Each group was exposed to 10 h of natural light (between 08:00–17:59 h) and 6 h of LED light (between 18:00–23:59 h) in the compartments. For 7 weeks, the first week of which was planned as an adaptation, the birds were exposed to coloured LED lights (white 400–740 nm, blue 450–490 nm, yellow 560–590 nm, and red 635–700 nm for 6 h/day) in such a way as to have 10 lux of light intensity at the base of the cage (Figure 1) [49].

The illumination periods (bright setting from sunset to midnight) of the atmosphere (home or office) where domesticated budgerigars are housed by the breeders were used as examples to determine the intensity and application time of the coloured lights used in the study. Moreover, white light, which budgerigars are more exposed to in the home and office settings, was assigned as the control group in the present study. The light intensity was measured at the base level of the cage at least once a week for 7 weeks using a digital lux meter (Extech5-in-1 Environmental Meter EN300).

### 2.3. Behaviour Observations in the Home Cage

**Figure 2:**
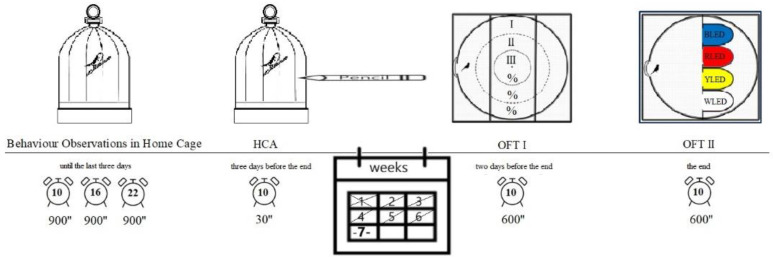
Timeline showing when the behavioural testing occurred during the study.

Behavioural observations that did not disturb the birds were used in the study. Behavioural observations were made three times a day (10:00 am, 4:00 pm, and 10:00 pm) at least 15 min after entering the laboratory using the time sampling method (Figure 2). The same observer made the observations each time, sitting at least 1 m away from the cages. The observer wore a white apron throughout all observations. Any action that might compromise the observation was avoided, and clothes with noticeable colours were not worn. The observer applied no perfume throughout all observations [50]. Observations were made according to the criteria set out in Table 3. During the observation, the birds’ feeding, comfort, resting, and social behaviours were assessed [51].

### 2.4. Home Cage Avoidance

The reactions of the birds to an item approaching them were observed using this test. The birds were not released from their cages during the test. Before the test, necessary precautions were taken to ensure that the birds would not be able to see the object (pencil) used for the test. In this context, the researcher came in front of the bird’s cage and extended the same pencil, which had been determined earlier, into the cage with the right hand, keeping it steady for 30 s, and the reactions of the birds were assessed based on the criteria set out in Table 4 [52].

### 2.5. Open Field Tests (OFTs)

**Figure 3:**
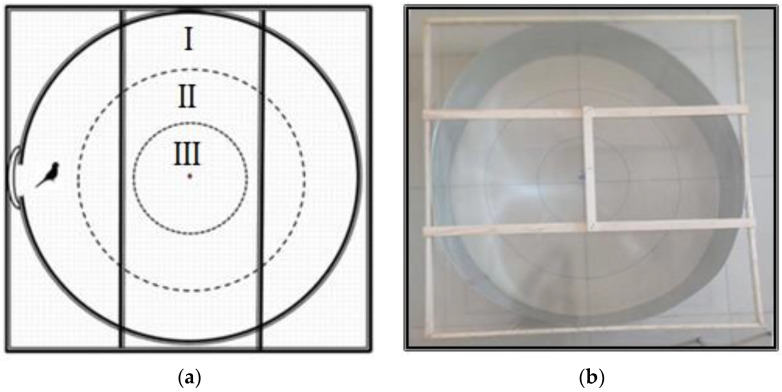
Behavioural analysis platform 1 (BAP I) for OFT I ((**a**) diagram, (**b**) photo). I, edge of the platform; II, middle of the platform; III, centre of the platform.

OFT I and II were performed in different controlled research rooms with the same breeding conditions (25 ± 2 °C temperature, 55 ± 5% relative humidity, and 35–40 dB noise level) where the birds were raised throughout the experiment. The behavioural analysis platform 1 (BAP-I) designed for this work was used for OFT I (Figure 3). The analysis platform was built by modifying a platform used in previous experiments [53]. The birds’ interest in an unfamiliar object they were thought to have never seen before was assessed throughout the test. Only one bird was transported in the same transport cage from the breeding habitat to the testing room for each test run. The bird was left inside the platform where the observation would take place after the 5 min adaptation period (Figure 3). The time spent by the birds in three equal parts of the platform for 10 min from the top of the platform was calculated, and their interest in the unfamiliar object hanging in the centre was assessed.

**Figure 4:**
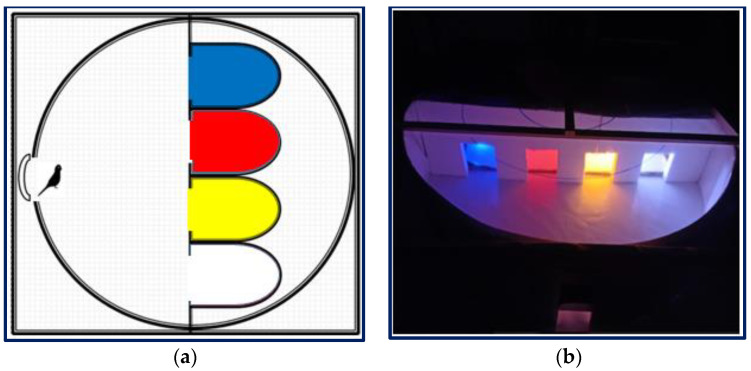
Behavioural analysis platform 2 for OFT II ((**a**) diagram, (**b**) photo).

OFT II was performed using the BAP-II platform (a platform built for this study), which was modified from a previous experiment (Figure 4) [54]. In the test, the interest and preference of birds for familiar and unfamiliar environments presented together were evaluated.

### 2.6. Statistical Analyses

After the data were obtained, the Kolmogorov–Smirnov normality test was performed, and it was determined that the data were normally distributed. A one-way analysis of variance ANOVA was used to determine some of the behavioural responses of the budgerigars raised in cages enriched with LED lights of different wavelengths [55]. The dependent variables were the behavioural responses (behaviour observations in the home cage, HCA, and OFT I and II) and food and water intake. The independent variables were the coloured LED lights (WLED, BLED, YLED, and RLED). The time expended in 600 s for the edge, middle, and centre of BAP I was recorded relative to the total time. The number of birds that entered the coloured compartments of BAP II in a maximum of 600 s was recorded. The OFT I and II outcomes were calculated as percentages. In the circumstances where the differences were determined to be significant, Duncan’s test, one of the multiple comparison tests, was performed to identify which exposure(s) produced this difference. The data were all distributed normally. The level of significance in the study was determined at *p* < 0.05. The analysis process used the analysis software SPSS (IBM SPSS Statistics 26; IBM-SPSS Inc., Chicago, IL, USA).

## 3. Results

### 3.1. Food and Water Consumption

The amount of daily food (g/bird) and water consumption (mL/bird) per bird are shown in Table 5. The mean amount of daily food consumption per bird was similar in all groups, and according to Duncan’s multiple comparison test, no statistical difference was found (*p* = 0.726). Likewise, the mean live weight at the end of the experiment was 43.47 ± 5.89 for all groups (*p* > 0.05). The mean amount of daily water consumption per bird (g/bird) was determined to be 8.30 ± 2.22 mL, 4.443 ± 1.86 mL, 3.63 ± 2.18 mL, and 7.81 ± 1.64 mL in the control, BLED, YLED, and RLED groups, respectively (*p* = 0.001).

### 3.2. Behaviour Observations in the Home Cage

**Figure 5:**
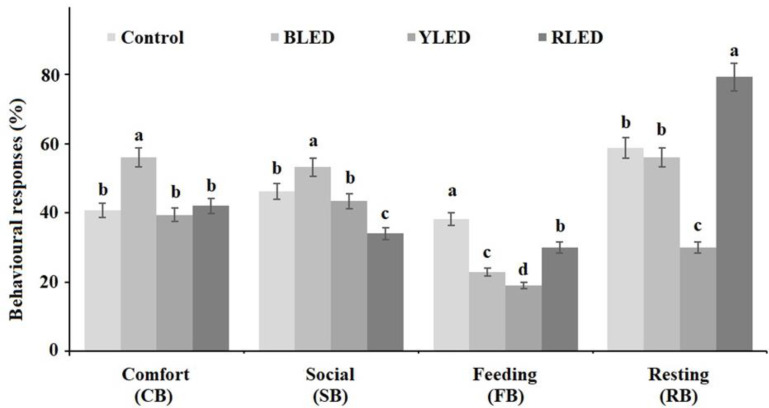
Comfort, social, feeding, and resting behaviours of the groups in their cages. a, b, c-different lowercase superscripts were significantly different at *p* < 0.05.

The differences in observations were found to be statistically significant at the level of *p* < 0.05. Figure 5 shows the results. In the study, the control group of birds displayed mostly feeding behaviours in their cages. The BLED group of birds showed more comfort and social behaviours. The RLED group of birds spent most of their time resting.

### 3.3. Home Cage Avoidance

**Figure 6:**
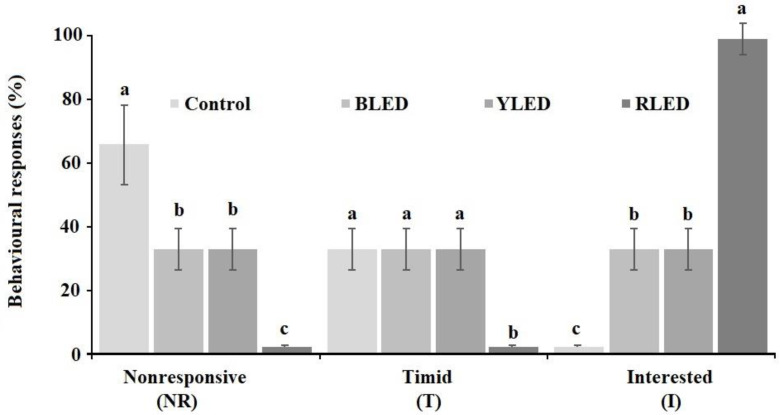
Home cage avoidance results (%). a, b, c-different lowercase superscripts were significantly different at *p* < 0.05.

Figure 6 shows the groups’ home cage avoidance results. The differences between the groups were found to be statistically significant at the level of *p* < 0.05. It was observed that the RLED group displayed a high response to the stimulus. The lowest response was found in the control group.

### 3.4. Open Field Tests

**Figure 7:**
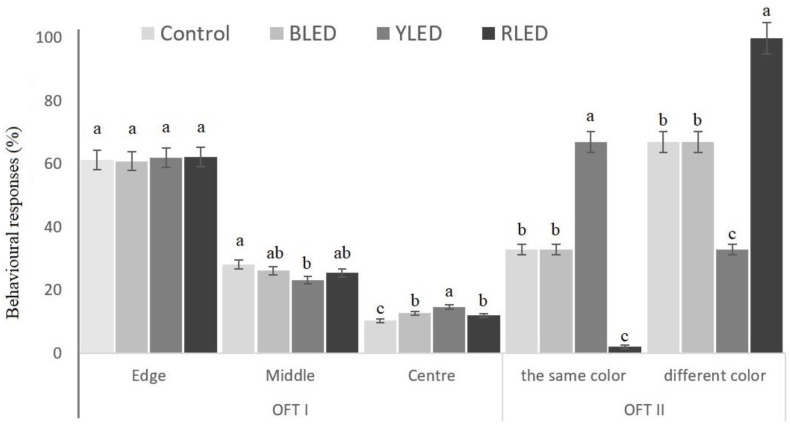
Behavioural responses of the birds to an object that they were thought to have never seen (unfamiliar object) in OFT I (positions of the birds on the platform %). The results of the OFT II test observation rate of preferring their own or a different coloured compartment (%). Means with different lowercase superscripts are significantly different at *p* < 0.05.

Figure 7 shows the results of the OFT I test for budgerigars housed under LED lights of different wavelengths. The differences between the groups were statistically significant at the level of *p* < 0.05. The RLED group received the highest score in compartment I, the control group had the highest score in compartment II, and the YLED group obtained the highest score in compartment III, where an unfamiliar object was hung in the centre of the platform.

The differences between the groups were statistically significant at the level of *p* < 0.05. It was observed that the birds of the YLED group preferred the compartment enriched with yellow light in the OFT II test.

## 4. Discussion

Many studies have shown that some behavioural disorders are associated with in-creasing light pollution worldwide [56,57,58]. In the present study, the treatment groups of birds housed with different coloured lights behaved differently. The possible biological explanations for this and what this means for the budgerigar’s wellbeing are discussed. The daily food intake was reported to be 2.6–6.4 g/bird in previous studies [59,60,61]. The food consumption of 4.62–5.14 g/bird, which was found in the present study, was within the results of previous studies. The range might be associated with differences in the live weights of the birds used in other studies, as well as differences in the care and feeding conditions.

Table 5 shows the daily water-consumption amounts (ml/bird) for the groups. The differences between the groups were statistically significant at the level of *p* < 0.05. The control group had the highest water consumption (8.30 mL/bird), whereas the YLED group consumed the least amount of water (3.63 mL/bird). That amount was between 3.86 and 4.5 mL, and it increased due to the impact of artificial light at night [31]. Some studies have reported that drinking less water increases stress levels [62,63]. In addition, less water consumption may increase the cortisol level in the body and cause it to feel more stressed [64]. In this study, it is thought that those who drank more water (control and RLED) may have done so in an effort to reduce possible stress. The amount of daily water consumption determined in this study was higher than in some studies by MacMillen and Baudinette (1993) and Kasuya et al. (1985) [65,66], but it was similar to another one [31]. Water consumption is affected by external factors such as the temperature, humidity, illumination, and food consumption in the habitat of the birds, as well as factors associated with the bird (age, sex, etc.).

Several studies on birds indicated that blue light calmed them, induced more sleeping and sitting behaviours, and increased the frequency of feeding and wing/leg stretching movements. Yellow light increased their walking and activity. Red light increased activities such as walking, flapping, and wing/leg stretching, but exacerbated their aggressive behaviour [67,68,69,70,71]. In this study, it was observed that the budgerigars in the BLED group exhibited more comfort and social behaviours. The YLED group showed the lowest resting and feeding behaviours. This situation can be evaluated for birds with obesity problems (Figure 4). These behaviours were not aggressive (Figure 5). The RLED group showed the highest resting behaviour. However, in the HCA test, the most aggressive behaviour (interested) was also in the RLED group. This situation may then be a melancholic but aggressive expression of resting in the cage.

If careful observation is not undertaken during an HCA test, then interested behaviours may be confused with aggressive responses to the stimuli. Moreover, similar behaviours in animals may appear as a part of the fight-or-flight response. In addition to the animal’s response to a threat, recession and fear behaviour may be an indication of a stress response, and stress adversely affects the health and productivity of birds at varying levels [72]. Although the responses of the birds in the RLED group were considered interested behaviour, the behaviour of the birds in this group can also be considered as an aggressive response to a sudden stimulus. 

In the current study, the RLED birds avoided the red-coloured light compartment. They may not have wanted to be in an aggressive and sluggish mood, evidenced by their behavioural tests. When presented with a choice, they selected another area. We can say that the behaviour of birds is influenced by previous experiences. The YLED birds preferred the yellow-lighted compartment. Presumably, unlike the RLED birds, they were not aggressive, as Open Field Test II showed. Moreover, according to Open Field Test I, they were less anxious than the groups exposed to other colours of light.

## 5. Conclusions

This study revealed that different coloured lights played an important role in the behavioural and physiological responses of the budgerigars. In practice, we should consider the lighting factors with different coloured LED usage strategies from multiple aspects. However, there were limitations to this study; the corticosterone level in the blood of birds was not measured, the sample size (*n* = 12 birds) was small, the time frame (7 weeks) was short, and we did not include a group housed with no light in the evening (in addition to white, red, blue, and yellow). The connection between water consumption and stress should be evaluated. For caged birds, water should always be available. Therefore, water consumption and stress and yellow and blue light should be the focus of future studies. The findings of the present study will facilitate the development of an illumination system for animal welfare to improve the bird housing conditions. Finally, people can use the results of our study to improve the living conditions of their birds in captivity, as companion animals.

## Figures and Tables

**Table 1:** Water-analysis values (mg/L) utilized in the experiment.

F−	HCO_3_	ClO_2_^−^	SO_4_	Ca	Mg	K	Na	pH	TMM
0.096	86.9	4.1	2.74	19.37	4.02	<0.2	2.2	7.55	122

F^−^, fluoride; HCO_3_, bicarbonate; Cl, chlorine; SO_4_, sulphate; Ca, calcium; Mg, magnesium; K, potassium; Na, sodium; pH; TTM; total mineral matter.

**Table 2:** Dietary nutrient levels (dry-matter basis, %).

CP	CF	M	CF	CA
10.4	8.9	8	6.6	3.3

CP, crude protein; CF, crude fibre; M, moisture; CF, crude fat; CA, crude ash.

**Table 3:** The ethogram used to observe the behaviours of budgerigars in their cages.

Behavioural Classification	Physical Activity
Feeding	Eating or drinking water
Comfort movements	Stretching the wings, stretching the legs, scratching the head with the foot, preening the feet, and preening the feathers
Resting	Perching, sleeping, and yawning
Social behaviours	Singing, flirting, clinging to the cage bars, trembling, and ruffling feathers

**Table 4:** The ethogram used to observe the behaviours of budgerigars in their cages for HCA.

Behavioural Classification	Physical Activity
Non-responsive	Those who remain calm and non-responsive
Those who are timid	Those who move to the other side of the cage
Those who are interested	Those who try to examine the shown object without fleeing, or peck unexpectedly

**Table 5:** Daily food (g/bird) and water consumption (mL/bird) per bird.

	DFI	SE	DWC	SE
Control	4.83	1.17	8.30 ^a^	2.22
BLED	4.62	1.59	4.43 ^b^	1.86
YLED	5.29	1.44	3.63 ^b^	2.18
RLED	5.14	1.36	7.81 ^a^	1.64
SEM	0.286		0.340	
F	3.643 ^NS^		21.319 ^S^	
*p*	0.726		0.001	

DFI, daily food intake (g/bird/day); DWC, daily water consumption. WLED (control): the group was exposed to white LED light (400–740 nm), representing today’s human home environment. BLED: the group was exposed to blue LED light (450–490 nm). YLED: the group was exposed to yellow LED light (560–590 nm). RLED: the group was exposed to red LED light (635–700 nm). The values are expressed as the means. Means with different lowercase superscripts are significantly different at *p* < 0.05. S, significant; NS, non-significant.

## Data Availability

The data are contained within the article.
